# Evaluation of Inhibitory Activity In Silico of In-House Thiomorpholine Compounds between the ACE2 Receptor and S1 Subunit of SARS-CoV-2 Spike

**DOI:** 10.3390/pathogens10091208

**Published:** 2021-09-17

**Authors:** Victor H. Vázquez-Valadez, Alejandro Hernández-Serda, Ma. Fernanda Jiménez-Cabiedes, Pablo Aguirre-Vidal, Ingrid González-Tapia, Laura Carreño-Vargas, Yoshio A. Alarcón-López, Andrea Espejel-Fuentes, Pablo Martínez-Soriano, Miguel Lugo Álvarez, Ana María Velázquez-Sánchez, Nathan Marko Markarian, Enrique Angeles, Levon Abrahamyan

**Affiliations:** 1 Departamento de Ciencias Biológicas FES Cuautitlán, Universidad Nacional Autónoma de México (UNAM), Av. 1 de Mayo SN Cuautitlán Izcalli, Estado de México, México CP 54750, Mexico; hugounam83@gmail.com; 2 Departamento de Ciencias Químicas FES Cuautitlán, Universidad Nacional Autónoma de México (UNAM), Av. 1 de Mayo SN Cuautitlán Izcalli, Estado de México, México CP 54750, Mexico; asdfgfamail@gmail.com (A.H.-S.); arturin_sirio@yahoo.com.mx (P.M.-S.); velzquezanamara@gmail.com (A.M.V.-S.); angeles@unam.mx (E.A.); 3 Laboratorio de Química Medicinal y Teórica FESC, Universidad Nacional Autónoma de México (UNAM), Av. 1 de Mayo SN Cuautitlán Izcalli, Estado de México, México CP 54750, Mexico; feanda980420@gmail.com (M.F.J.-C.); pyogenes2heli@gmail.com (P.A.-V.); ingridrew1@gmail.com (I.G.-T.); lauracarrevar@gmail.com (L.C.-V.); yoshalar@gmail.com (Y.A.A.-L.); andrea.espejel95@gmail.com (A.E.-F.); mlugoa@gmail.co (M.L.Á.); 4 Swine and Poultry Infectious Diseases Research Center (CRIPA) and Research Group on Infectious Diseases in Production Animals (GREMIP), Faculty of Veterinary Medicine, University of Montreal, Saint-Hyacinthe, QC J2S 2M2, Canada; nathan.marko.markarian@umontreal.ca

**Keywords:** SARS-CoV-2, thiomorpholine derivatives, spike, antivirals

## Abstract

At the end of 2019, the world was struck by the COVID-19 pandemic, which resulted in dire repercussions of unimaginable proportions. From the beginning, the international scientific community employed several strategies to tackle the spread of this disease. Most notably, these consisted of the development of a COVID-19 vaccine and the discovery of antiviral agents through the repositioning of already known drugs with methods such as de novo design. Previously, methylthiomorphic compounds, designed by our group as antihypertensive agents, have been shown to display an affinity with the ACE2 (angiotensin converting enzyme) receptor, a key mechanism required for SARS-CoV-2 (severe acute respiratory syndrome coronavirus 2) entry into target cells. Therefore, the objective of this work consists of evaluating, in silico, the inhibitory activity of these compounds between the ACE2 receptor and the S1 subunit of the SARS-CoV-2 spike protein. Supported by the advances of different research groups on the structure of the coronavirus spike and the interaction of the latter with its receptor, ACE2, we carried out a computational study that examined the effect of in-house designed compounds on the inhibition of said interaction. Our results indicate that the polyphenol LQM322 is one of the candidates that should be considered as a possible anti-COVID-19 agent.

## 1. Introduction

In December 2019, a new type of disease has been reported in the city of Wuhan (Hubei Province, China), where many cases of atypical pneumonia were diagnosed [1]. The causative agent of this disease was soon determined to be due to a novel coronavirus, initially named “2019-nCoV”and known today as severe acute respiratory syndrome coronavirus 2 (SARS-CoV-2). With the rapidly increasing number of cases and deaths, coronavirus disease 19 (COVID-19) spread to several countries and was declared a pandemic by the World Health Organization on 11 March 2020 [2]. Although sanitary measures have been implemented by several countries to combat infection rates, such as social distancing, quarantine, contact tracing, and testing, COVID-19 has claimed more than 4 million lives and has been responsible for approximately 200 million cases worldwide as of 11 July 2021 [3]. The emergence and expansion of SARS-CoV-2 has indeed caused immeasurable damage to the global health and economy sectors, which is why it is of great importance for all countries to collaborate to fight against this deadly disease.

The novel coronavirus, SARS-CoV-2, is a capped single stranded positive sense RNA (ssRNA) coronavirus that belongs to the *Betacoronavirus* genus [4] of the *Sarbecovirus* subgenus, part of the *Coronoviridae* family of the *Nidovirales* order. Like other coronaviruses, its genome is about 30 kilobases (kb) in length and contains a 5′ untranslated region (UTR) and a 3′ poly(A)-tail at the 3′ UTR, allowing it to have a similar structure to the host cell mRNAs [5,6,7]. Besides both 5′ and 3′ UTRs, its genome also contains the overlapping ORF1a and 1b genes that encode 15–16 non-structural proteins (nsps), many of which assemble into a replication/transcription complex (RTC) [8]. Furthermore, viral structural proteins, including the spike (S), envelope (E), membrane (M), and nucleocapsid (N), as well as other accessory proteins, are encoded in the genes downstream of ORF1a/ORF1b [9].

SARS-CoV-2 virions are enveloped and spherical, measuring around 100 nm in diameter [10]. Three transmembrane proteins are incorporated in the viral lipid bilayer, including the E, M, and S proteins, and the ssRNA genome is packaged within a helical nucleocapsid made of nucleocapsid (N) proteins [11,12,13]. The major surface glycoprotein of SARS-CoV-2 is the spike (S) protein, as it is responsible for binding to host cell receptors, mainly to the dipeptide carboxypeptidase angiotensin converting enzyme (ACE2) [14]. The latter is found in various sites susceptible to infection, such as in lung alveolar epithelial cells and small intestine enterocytes [15]. The SARS-CoV-2 spike protein has a homotrimeric structure of which each monomer (180 kDa, 1273 amino acids) contains two subunits: S1 (14–685 AA), which is the most variable region, and S2 (686–1273 AA), which is conserved by the structure and sequence [16,17]. In the S1 subunit, there is an N terminal domain NTD (14–305 AA), receptor binding domain RBD (319–541 AA), and a receptor binding motif RBD (437–508 AA). As for the S2 subunit, it contains a fusion peptide FP (788–806 AA), Heptad Repeat 1 HR1 (912–984 AA), Heptad Repeat 2 HR2 (1163–1213 AA), transmembrane domain TM (1214–1237 AA), and a cytoplasm domain CP (1238–1273 AA) [18]. To enter the target cells, SARS-CoV-2 virions bind to the peptidase domain (PD) of ACE2 via the receptor binding domain (RBD) of its S1 protein, and this is followed by an essential acid-dependent cleavage at the S1/S2 site, which can be achieved by proteases such as the cellular serine protease TMPRSS2 [19,20]. The latter process triggers an additional cleavage of the S2 subunit at the S2′ site, which allows it to expose the fusion peptide, and with conformational changes, the virus can fuse with the host cell membrane [21]. 

As the number of COVID-19 cases continue to rise, especially in countries where there have not been any sanitary measures put in place, there is an accumulation of mutations in circulating SARS-CoV-2 strains, which allows for the possibility of new variants. A prime example of this is the D614G single nucleotide polymorphism (SNP), which appeared in March 2020 and subsequently became the dominant haplotype worldwide, as it renders the virus more infectious [22]. Several other mutations have been observed in the context of variants, many of which have been identified since January 2021. Of these, some are known as variants of concern (VOC), namely alpha (B.1.1.7), beta (B.351), gamma (P1), and delta (1.617.2), and have been noted to possess increased transmissibility, where some increase disease severity and others have shown a potential to reduce post-vaccination sera neutralization [23,24,25]. 

During the ongoing COVID-19 pandemic, several strategies to control the spread have been implemented, the most successful being vaccination [26]. However, with SARS-CoV-2 variants on the rise, the efficacy of the available vaccines can potentially be reduced, allowing the virus to spread further. Because of this, many groups of scientists around the world are currently working hard to find, through different methodologies, an arsenal of effective drugs against the virus. One such strategy is drug repositioning, which consists of testing already-approved drugs used for other diseases against SARS-CoV-2 [27,28,29,30]. In addition to this, various research groups have used their own molecules synthesized in their laboratories and have proposed them as possible candidates against COVID-19 [31,32]. 

To this day, there are several reports where the interaction between the S1 spike subunit and ACE2 has been resolved, indicating the specific regions of interaction and the impact of the corresponding mutations on that same interaction [33,34,35,36]. A potential drug candidate against SARS-CoV-2 would aim to weaken this interaction, thus preventing viral entry into target cells. In a previous study, we obtained experimental and computational data after investigating the effects on ACE1 using five inhibitors synthesized in our laboratory, belonging to a family of 45 compounds known as LQM (Table 1). These molecules had different degrees of inhibition to ACE1, where the order from the most favorable compound to the least was as follows: LQM322, LQM319, LQM324, LQM318, and LQM304 [37]. Before proceeding, an identity analysis between ACE1 and ACE2 was performed. The percent identity between both proteins was 21%, and this was considering the complete sequence of both proteins. Furthermore, an analysis of the pocket where the most favorable affinity for the LQM compounds was also carried out. This comparison of the identity of the binding region considered a radius of 9 Å, and the percentage of identity between ACE1 and ACE2 in the studied zone was 55.4%. With this identity percentage, the results could not be extrapolated, but there was an adequate level of identity to do an independent study in ACE2, and by having ACE1 as the background and not as a template. Given these results, we herein computationally investigated the interaction of ACE2 with these inhibitors (referred as ACE2–In), as well as the ACE2–In interaction with the S1 subunit of the SARS-CoV-2 spike (referred as S1–ACE2–In). From the tested compounds, LQM322 was shown to significantly decrease the interaction between ACE2 and the S1 viral spike subunit. This inhibitory effect can be further tested in future studies in vitro, serving as a starting point for the study of its use as a possible antiviral. This work is currently in progress in our laboratory (Dr. L. Abrahamyan, Faculty of Veterinary Medicine, University of Montreal).

## 2. Results and Discussion

### 2.1. Validation Process of Molecular Protein–Protein Coupling

**Figure 1:**
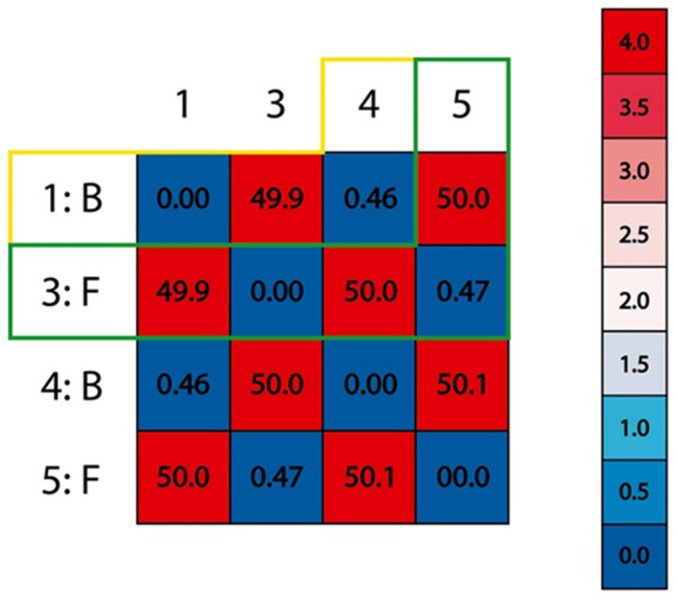
RMSD (root-mean-square deviation) of each component of the system, in yellow boxes, chain 1 and 4 belonging to ACE2 are compared with a 0.46 Å deviation, and for the green boxes, chains 3 and 5 belonging to the spike are compared with a 0.47 Å deviation.

Once the molecular docking for the protein–protein system was completed, the most favorable energetically and geometrically positions were used. For this case, it was found that the position calculated for the spike differed from the experimental position by 0.47 Å. Additionally, for the position calculated for ACE2, the latter differed from the experimental position by 0.46 Å (Figure 1). With this result, the protocol used for the molecular recognition process was suggested to be adequate for this system.

### 2.2. Molecular Coupling Protocol and MOE-NAMD (Molecular Operating Environment-Nanoscale Molecular Dynamics)

In this section, the systems were analyzed together as the molecular dynamics were carried out from complexes calculated from molecular coupling. In molecular dynamics, processes that refer to ACE2–In complexes, the RMSD (root-mean-square deviation between specified sets of atoms), RMSF (root mean square fluctuation), and the total hydrogen bonds of the protein were all analyzed during simulation, as well as the hydrogen bonds formed between the ligand and the protein, and the free energy of binding. These procedures were calculated with visual molecular dynamics (VMD). The distances between ASN63 and ASN121 were also measured to analyze the behavior of ACE2 in the context of LQM inhibitors. Amino acids ASN63 and ASN121 were used, as each of them belongs to a region that is known to be adjacent when ACE2 acquires a closed conformation. This was therefore done to be able to carry out a geometrical analysis of ACE2 and to see if this affects the interaction with the S1 fraction of the viral spike.

**Figure 2:**
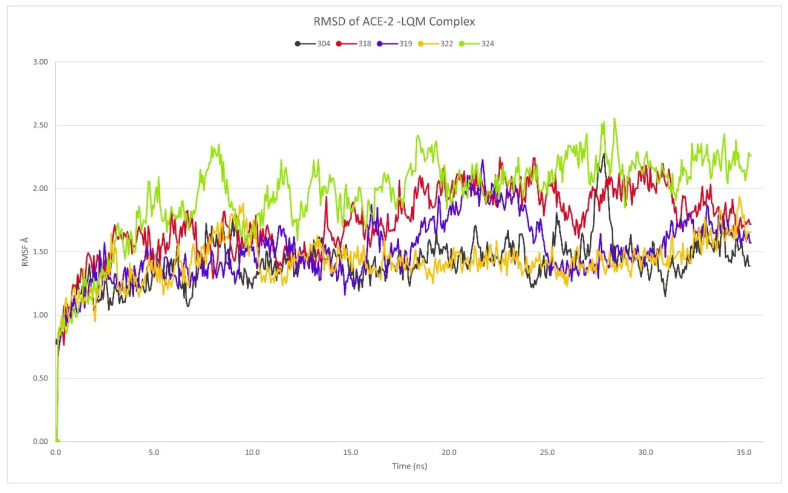
RMSD calculated from studied complexes ACE2–LQM304, ACE2–LQM318, ACE2–LQM319, ACE2–LQM322, and ACE2–LQM324.

The RMSD in all cases showed that the dynamics simulation reached a stability for every complex, as there were no deviations that suggested instability in this parameter. In the cases studied, RMSD did not exceed 2.5 Å and in a particular case of LQM322, it did not exceed 2 Å (Figure 2).

**Figure 3:**
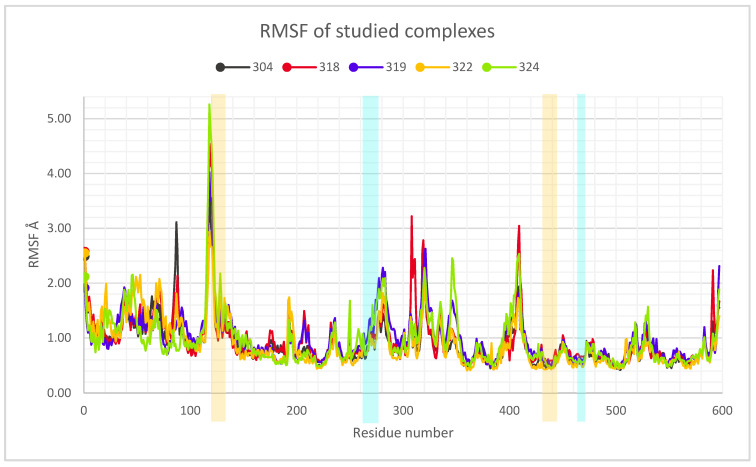
RMSF of the studied complexes of each amino acid on ACE2 during ACE2–inhibitor simulations.

Another calculated parameter was the RMSF, as shown in Figure 3. When a dynamic system fluctuates around a well-defined average position, the RMSD of the average over time can be called RMSF (root mean square fluctuation). In other words, the RMSF was calculated to determine the level of fluctuation of each amino acid, and thus to identify if, within the dynamics, there would be any anomaly that would have suggested some important change. Usually, this parameter should not be analyzed in an isolated fashion and other parameters are needed to determine the level of fluctuation or possible instability in the system. In the case of the systems studied, the RMSF did not present important fluctuations that would have suggested that the systems were destabilized or that they lost their structural integrity. The most important fluctuation levels were around residues 120, 275, 325, and 400. All of these fluctuations are from exposed regions and do not have a defined geometry and lack of stability as a helix or a beta sheet structure. That is, they are exposed loops and turns. Furthermore, each of the RMSF fingerprints was consistent between all of the proteins of each of the systems studied. The RMSF plot of the systems are presented below in Figure 3, which are also indicative of the protein integrity or possible-protein destabilization. The residues highlighted in Figure 3 in the orange boxes are those mentioned above that are in high mobility regions of the protein. On the other hand, the residues that are in the blue boxes are the residues that are in interaction with the proposed inhibitors. RMSF corroborates that the residues are not fluctuating in a way that suggests instability in the systems, that is, if the RMSF does not increase, this indicates that the residues do not have such a free movement, which may be reduced by the interaction with the inhibitors. Unlike compound LQM324, in the case of LQM324, an increase in the RMSF parameter of the interaction residues is seen. This behavior indicates that the residues in the region are not stabilizing their mobility with the inhibitor. This can be corroborated with H-bonds. It is worth mentioning that in the calculation of the RMSF, there is an anomaly between residue 305 to 315 of the ACE2–LQM324 complex. This specific region belongs to an exposed area of the protein and the inhibitor is not near the mentioned region. This behavior is anomalous with respect to the rest of the systems and may be due to a vibrational state that increases the fluctuation of the zone or, less likely, an effect caused by the ligand, as the LQM324 compound is the only one with a nitro group. On the other hand, this compound did not show a favorable affinity, so a more in-depth analysis was not carried out.

**Figure 4:**
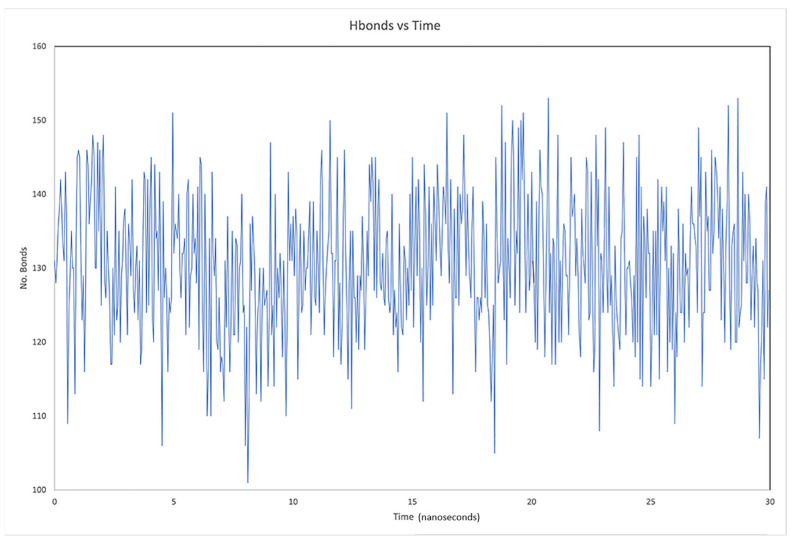
Hydrogen bonds of the ACE2–LQM322 system studied through the time of the dynamics.

Another way to verify the stability of the protein during dynamics, as mentioned before, is by counting the interstructural hydrogen bonds of each protein. That is, if the number of hydrogen bonds decreases, it may indicate a structural denaturation of the macromolecule during the dynamics, and this may be due to various factors. For example, if the internal energy increases, structural destabilization is almost imminent, and this is seen in the amount of hydrogen bonds [38,39]. According to this parameter, hydrogen bonds were identified in the study cases, and it was not found that there was a destabilization of any protein. In Figure 4, only the production state is shown. This figure also shows the behavior of the protein and hydrogen bonds only for the ACE2 in complex with LQM322. The hydrogen bonds during the MD simulation are consistent as there is no diminution of this parameter. Therefore, this suggests that the internal integrity of ACE2 is sustained during the calculations.

**Figure 5:**
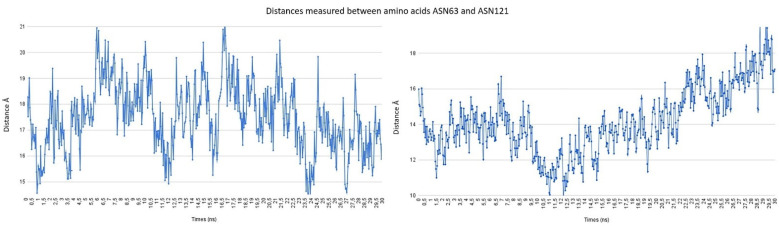
Distance between ASN63 and ASN121 of ACE2–LQM304 (**left**) and ACE2–LQM322 (**right**) systems.

The distances measured between amino acids ASN63 and ASN121 are shown in Figure 5. As depicted, both systems maintain a large distance between the residues. In general, even for LQM-304, the distance is greater than for LQM-322, indicating an initial more open conformation of the former system than the latter. The behavior of molecular dynamics suggests that for LQM-322, the distance increases as the simulation goes forward.

Although a 30 ns simulation is not sufficient to conclude whether LQM-322 keeps a more open conformation better than LQM 304, and that further escalation in simulation is needed, in general, both ligands can keep a distance between the above-mentioned amino acids ranging from 14 Å to 19 Å, showing that the open conformation of the protein is maintained by LQM compounds. 

To measure the interaction during the dynamic studies between inhibitors and protein, the hydrogen bonds between each ligand and ACE2 were quantified. This is a parameter that allowed us to quantify whether the ligand (the experimental compound) maintained an interaction with the protein (ACE2), in addition to the fact that the longer these interactions last and the greater the number of hydrogen bonds, it can be asserted that the interaction is more favorable.

**Figure 6:**
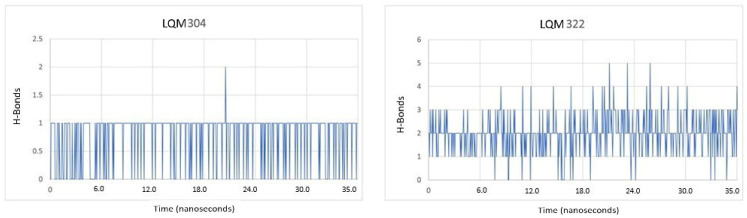
Hydrogen bonds present between the LQM304 (**left**) and LQM322 (**right**) compounds and ACE2 during molecular dynamics simulation.

As shown in Figure 6, the complex with LQM304 had the least hydrogen bonds. During the simulation time, only one bridge persisted. On the other hand, LQM322 continued fluctuating and had the most hydrogen bonds, but remained between two and four hydrogen bonds. These hydrogen bonds were present due to the number of centers that the LQM322 compound had. In comparison, the LQM304 compound had only two centers able to form hydrogen bonds with the hydroxyl of the benzene ring. On the other hand, the LQM322 compound had three hydroxyl groups of the polyphenol and the sulfur of the thiomorpholine that could form hydrogen bonds. However, hydrogen bonds are only one way to evaluate the possible complementarity that it will have for the receptor, and it is not possible to assert any conclusion with only this parameter. It must therefore be analyzed along with other parameters such as RMSD, RMSF, and free binding energy. At this point, the duration of the simulations was considered to be adequate to conclude that a dissociation of the compounds did not occur during the calculations, and the H-bonds remained constant. On the other hand, the latter needs to be kept in mind as, if these hydrogen bonds decreased during the simulation of MD, it could indicate, after reviewing the other parameters as mentioned, that at the same time, that the ligand lacked affinity with the receptor. From the previously reported experimental point of view, LQM322 presented the highest inhibitory activity compared with the other compounds studied.

Despite the advancement in computational technology, the accurate prediction of the binding affinities (free binding energies) of the protein–ligand interaction remains a very complicated and challenging task. Inaccurate prediction of the protein–ligand affinities could be due to different types of errors, such as incorrectly predicted binding patterns, lack of consistent binding data obtained using different assay methods and conditions, and flaws in computational methods. This is why it is necessary to carry out complementary studies that will allow us to have an adequate interpretation or to extrapolate the results obtained in this type of calculation. In the case of the present work, BFEE2 was used to estimate the free energy [40]. The Binding Free Energy Estimator (BFEE2) is Python-based software that automates absolute binding free energy calculations through the alchemical or geometric pathway using molecular dynamics simulations. The degrees of freedom of the protein–ligand (or host–host) system are described by a series of geometric variables (or collective variables), as first described by the Karplus group. In BFEE, generalized geometric variables based on the best fit rotation are used, which, in principle, is available for any protein–ligand complex. The results for each system are shown in Table 2. Both LQM322 and LQM319 showed the best behavior in the case of the evaluation of the calculated energies, where the least favorable were LQM314 and LQM304. In agreement with these results, the behavior seemed to remain consistent in accordance with the results obtained in the study previously carried out on ACE1.

### 2.3. Description of the Dynamics of Protein–Protein Systems

**Figure 7:**
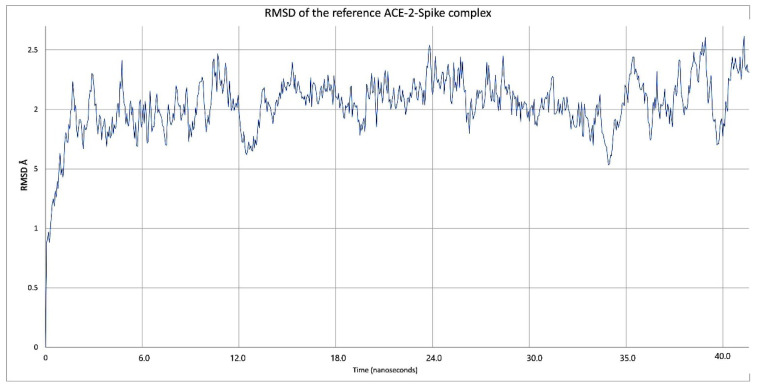
RMSD of the reference ACE2–spike complex during the 40 ns simulation.

Once the interaction and stability of each of ACE2–inhibitor systems had been studied, the same parameters were measured, except for the distance between amino acids ASN63 and ASN121 in the S1–ACE2–In complex. Initially, the RMSD of the ACE2–spike complex was measured, as this was the reference with which the studied systems were compared. Figure 7 shows the global graph of the RMSD of the ACE2–spike complex, and it can be concluded that the complex reached stability during the 45 ns that the simulation lasted.

**Figure 8:**
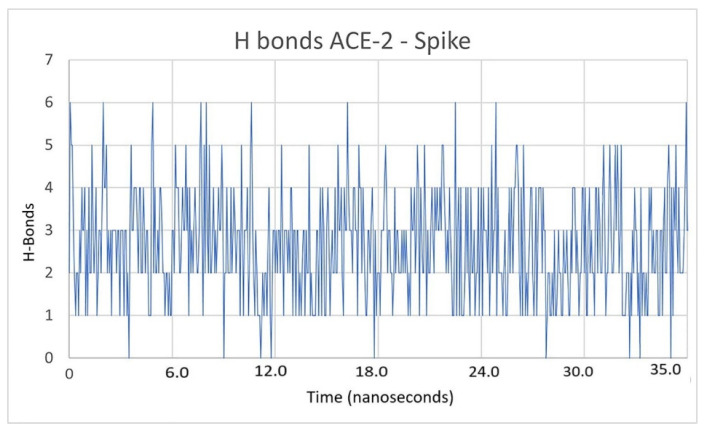
Hydrogen bonds present between ACE2 and the spike in the 45 ns simulation.

**Figure 9:**
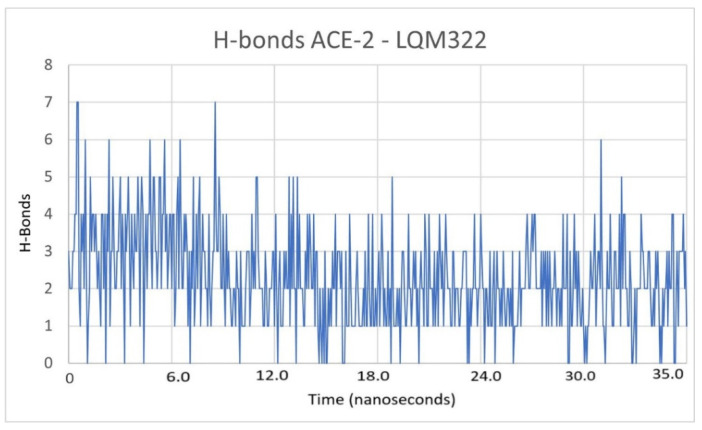
Hydrogen bonds present between ACE2–LQM322 and the spike in the 45 ns simulation.

**Figure 10:**
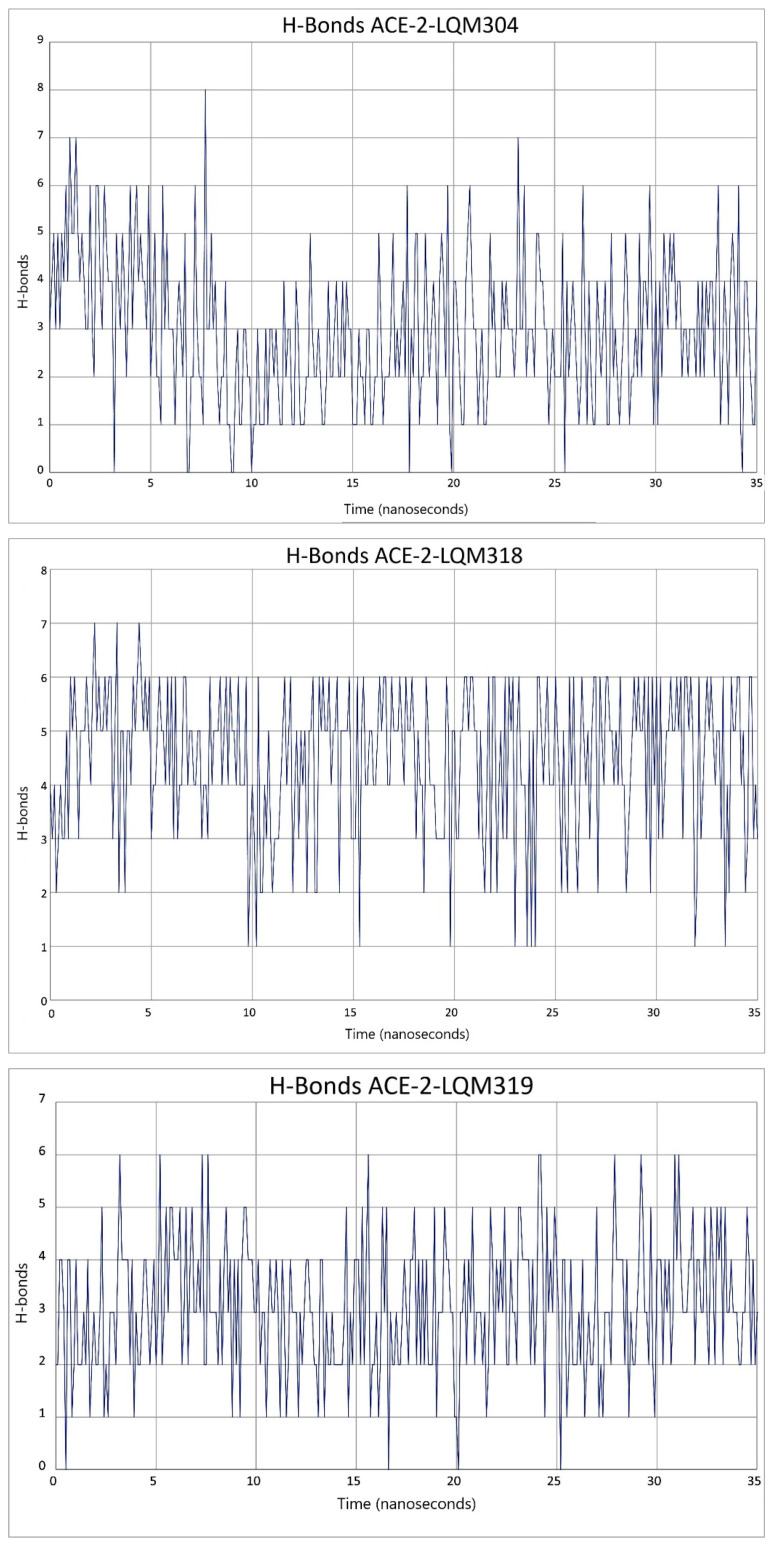

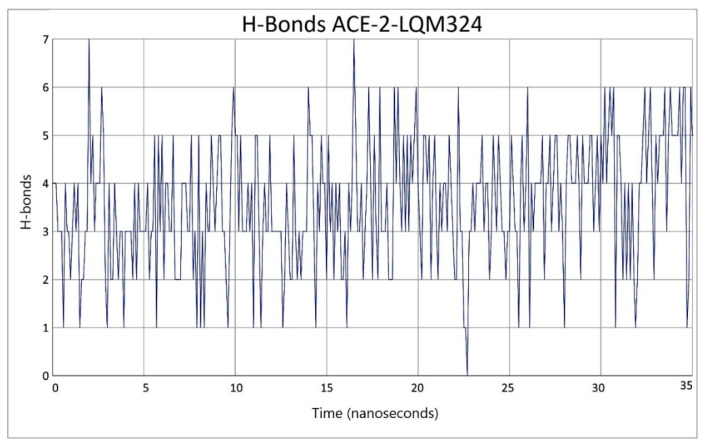
Hydrogen bonds present between ACE2–LQM compounds and the spike in the 45 ns simulation.

Regarding the hydrogen bonds that occurred between the ACE2 and the spike protein, there are also other parameters that must be considered, as they assess the stability of the interaction between both species. In Figure 8, hydrogen bond interactions were quantified, ranging between three and six hydrogen bonds. If inhibitors affected the interaction between ACE2 and spike in such a way that it destabilized the complex, then this parameter would decrease. As can be seen in Figure 9, the hydrogen bonds between spike and ACE2 were modified if an inhibitor was present, in this case it was LQM322. When these interactions were evaluated in presence of the latter, the hydrogen bonds decreased between two and three. Only half H-bonds from the reference structure were preserved. This may suggest a complex instability that could lead to a decrease in the interaction between the spike and ACE2 receptor [41]. The H-bonds calculated for each ACE2–LQM system were also calculated and are presented in Figure 10. To rule out that this behavior was due to a loss of structural stability for either of the two proteins, the interstructural hydrogen bonds of both proteins were computed and it was found that these interactions were maintained within a stable range, thus indicating that the structural stability of each species was maintained.

**Figure 11:**
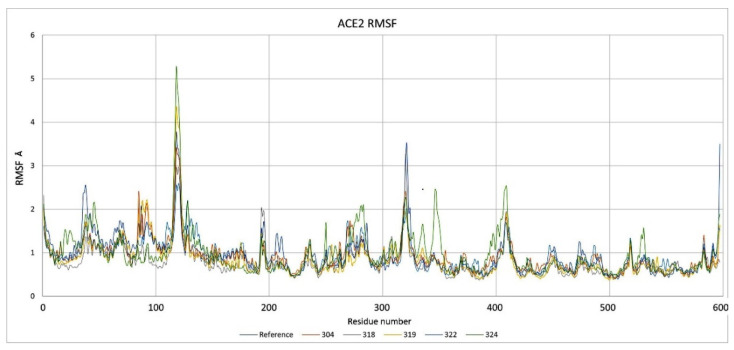
RMSF for the ACE2 protein during simulations in complex with subunit S1.

**Figure 12:**
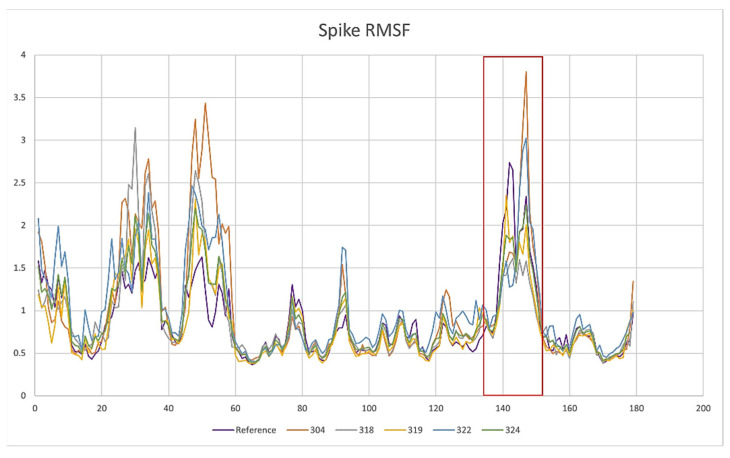
RMSF for subunit S1 during simulations in complex with ACE2; the red shade corresponds to the RBD from subunit S1.

Regarding the analysis of the results of molecular dynamics on ACE2–ligand–spike complexes, the RMSD parameters were also calculated for ACE2 and viral S protein, as well as the RMSF parameter (Figure 11). On the other hand, the fluctuations of the RMSF parameter for ACE2 did not suggest a significant alteration at the time of being evaluated. That is, ACE2 remained stable together with its corresponding inhibitor, preserving a stable interaction of the ACE2–inhibitor complex. However, the S1 subunit of the spike showed a fluctuation in the RBD. As shown in Figure 12, the marked region in the red box is the area where the RBD of S1 subunit from the viral spike was located. This region showed a difference with respect to the type of fluctuation that occurred with each inhibitor. Part of the RBD of the spike was found in the indicated region. This region had a low fluctuation in the reference system (ACE2 and spike). Fluctuations between residues 20 through 60 were the highly exposed regions of the spike. However, in the reference system, these regions were kept with less fluctuation compared with when the system incorporated the inhibitors. The RBD region was the most relevant as it presented the complementarity with the receptor. Fluctuations in this area were increased by inhibitors and this behavior indicates that there was not an adequate complementarity for the ACE2–spike complex to be maintained.

### 2.4. Procedure for the Analysis of “Patches” of the Interaction Surfaces

Patch analysis was performed to analyze the chemical complementarity [42] that existed in the spike S1–ACE2 and spike S1–ACE2 systems with inhibitors. Table 3 shows the results of the patch analysis on the SARS-CoV-2 spike S1 (ASA—accessible surface area) on the RBD of the spike. This includes the different types of patches, where each of these regions had their counterpart in the ACE2 receptor. Furthermore, the identification number of the patch is shown, followed by the amino acids present in each patch. The area in Å2 of each one of the patches and at the end the percent of the area with respect to the total surface area in Å2 is shown.

**Figure 13:**
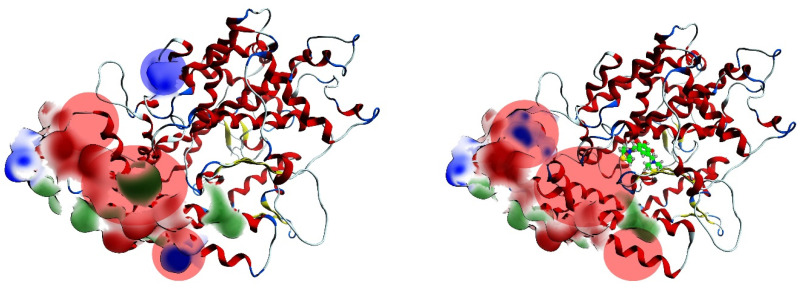
The patch analysis of the ACE2 (**right**) in absence and presence of LQM322: (**left**).

Moreover, Table 4 shows the interaction patches of the ACE2 and ACE2–inhibitor proteins. The table provides each contribution that was present in ACE2 without inhibitors and how it was affected with each of the inhibitors present. These patches were calculated from an average structure resulting from the dynamics performed. In other words, during the trajectory, the relative average positions were calculated to obtain these structures. Each of the inhibitors modified its respective patch in a time-dependent way, which affected the complementarity that existed between spike S1 and ACE2. Figure 13 shows the patches present in ACE2 with and without an inhibitor. The red patch indicates a negative electrostatic potential, the blue is positive, and the green is hydrophobic. The figure highlights the regions that were modified with the presence of the inhibitors from the average structure obtained from the ACE2–inhibitor dynamics. These modifications altered the molecular complementarity between the receptor and the viral spike. This may indicate why ACE2 could show a different behavior to spike S1.

## 3. Materials and Methods

The Molecular Operating Environment (MOE) 2019.01 program was used to carry out the computational studies [43]. Molecular dynamic simulations were performed with nanoscale molecular dynamics (NAMD) [44], and the visualizations and analyzes were carried out with visual molecular dynamics (VMD) [45]. 

For molecular dynamics calculations and docking studies, high-efficiency computer equipment was used, including an Intel Xeon Silver 4114 Dual processor computer with 20 cores, 40 4-GHz logic processors, 48 GB RAM, and Nvidia RTX2080 GPU. Another workstation had AMD Ryzen Threadripper 3960X at 3.9 GHz with 24 cores, 48 logic processors, 32 GB RAM, and Nvidia RTX2070 Super GPU and the “Miztli” supercomputer from Universidad Nacional Autónoma de México (UNAM). 

### 3.1. Preparation of Protein Structures

Initially, the complex of the SARS-CoV-2 S1 spike subunit interacting with human ACE2 (PDB ID 7KMB) was downloaded from the PDB (Protein Databank, rcsb.org) [46,47]. This complex was crystallized at a low pH 5, as occurs in the endosomal process, and was chosen for this study as the viral RBD was found to interact with the ACE2 receptor [47]. 

An analysis of the structural differences that occur at pH 5 and pH 7 was carried out to analyze the structural and energetic changes in the S1–ACE2 complex. For the preparation of the structures, the Molecular Operating Environment software was used and the topological corrections of the terminal regions of both structures were made. This was necessary because there were regions with a high mobility that, thanks to their low intermolecular interactions, tended to destabilize. Crystallographic data often have imprecisions at these terminal regions that are intrinsically highly mobile. In the same way, the partial charges of the system components were adjusted. The adjustment of the partial charges was carried out using the parameters of a ForceField (FF), in this case Amber10, included in MOE, which has the necessary parameter values of the partial and total charges of the atoms, as well as bonding geometry and nature.

Subsequently, the Protonate3D module was used, which aims to assign ionization states and place hydrogens atoms oriented according to the environment of a given system, with respect to the spatial coordinates. Most of the macromolecular structures obtained from the crystal contained little or no hydrogen coordinate data due to the limited resolution of the applied technique; however, the hydrogen bond network and the ionization state of the titratable groups could have a dramatic effect on simulation results. Specifically, this procedure was performed to describe and orient the hydrogen atoms throughout the system. Normally, the information input into the PDB lacks this information and only contains the heavy atoms. The protonation tools are also based on the parameters of the FF “Amber10: ETH”. For this calculation, the temperature was adjusted to 300 K and the pH was 5.0.

For the electrostatic function, Generalized Born formalism was used with integral volume formalism of GB/VI. The dielectric constant was adjusted to 80 as a solvated system was used. Once the charges, hydrogens, and terminal regions were adjusted, a slight refinement was carried out. This step allowed the structures to relax, minimize energy, and eliminate some steric effects that could modify the stability of the components, probably caused by the compaction of the protein in a crystalline state. The gradient used in this case was 0.1 kcal/mol/Å. This adjusted parameter allowed us a slight accommodation with the new components in the system, without causing significant structural or conformational modifications. To identify the RBD of the S1 spike subunit and the involved amino acids of ACE2, a pre-established set of amino acids was generated for each component. For S1, these were S109-T110 and P147-I166, and for ACE2, they were S1, T2, L6, T9, T13, Y16, E20, and G333-D337.

### 3.2. Validation Process of Molecular Protein–Ligand Coupling

To validate the molecular docking protocol, the procedure described in a previously performed study was carried out [48]. This was done to ensure that predictive models would not be affected by the structural differences caused by the different isoforms. Indeed, the protein family and system maintain conserved regions between the isoforms, and therefore, the same conditions were maintained. The conditions used were as follows: the triangle matcher method (optimized method for small or medium organic molecules) was used to search for the initial positioning of each of the ligands, and London dG formalism was used for the energetic evaluation. One hundred positions were returned from the orientations of the ligand at the possible site of interaction for the first evaluation. The grid assigned for protein–ligand docking was 10 Å, twice the size of the ligand in all directions, using wall restrains. Refinement was returned to 50 poses because when 100 were evaluated, no significant difference was found. The docking results were clustered based on the RMSD of the heavy atoms of the ligands with a maximum tolerance of 1 Å. The respective clusters of the docking resulting poses are shown in Supplementary Figure S1. Once this initial positioning process was finished, a refinement of the positions calculated was made. The returning processes were refined with an energy evaluation, using the GBVI/WSA dG formalism, returning 50 probable positions at the end of the calculation.

### 3.3. Validation Process of Molecular Protein–Protein Coupling

To validate the designed molecular docking protocol, the following procedure was performed: the macromolecular complex was separated into two independent files. One corresponded to the viral spike protein (ligand) and the other to ACE2 (receptor). For this specific process, only one subunit of the spike trimer was used. Each component of the already separated system underwent geometric and energetic optimization. For this part, a correction that considered all of the atoms was used, and the calculation of the partial charges was computed using Amber10 with EHT ForceField. The adjustment of the distances and angles of the OH groups were allowed to reach their optimal orientation. Water molecules remained as rigid bodies and the gradient used for minimization was 0.01 RMS Kcal/mol/Å2. To assign the calculation volume for protein–protein docking, a search box was assigned, defined as the center on the THR31 of the RBD of ACE2. The box had a geometry of 15 × 15 × 15 Å, establishing rotations of the amino acids of the targets with an RMSD of no greater than 2 Å. 

Once the components were prepared independently, the molecular docking was carried out using the Dock Protein–Protein module. The subsets of atoms used were as follows:

The receiver was ACE2, occupying the sets as a reconnaissance site.

**Figure 14:**
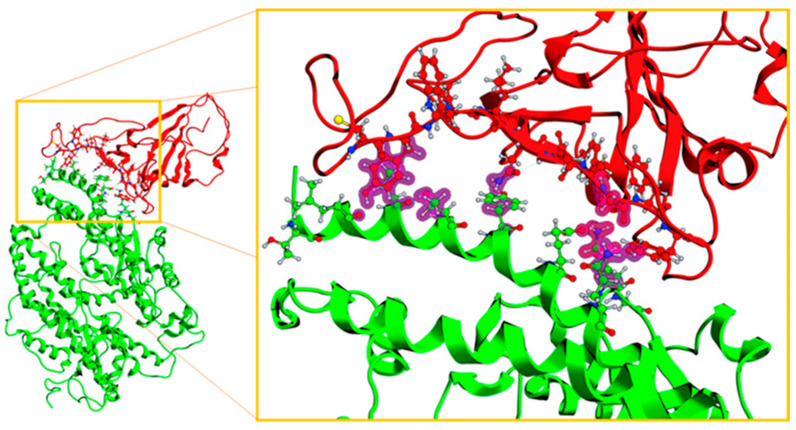
Complex of subunit S1 of the viral spike with the ACE2, with the representation of the viral RBD (receptor binding domain; red) and the ACE2 receptor amino acids involved (green).

For the ligand, the S1 subunit of the viral spike was assigned, and the recognition site was selected with respect to the possible interaction sites (Figure 14).

### 3.4. Molecular Docking Protocol

In this section there are different molecular docking processes. This is because of the different couplings, which were raised as follows: during virus entry, molecular recognition occurred first between ACE2 and RBD in the S1 region. This is what subsequently allowed the virus to enter the host cell. In this case, molecular coupling occurred between the receptor protein (ACE2) and the ligand protein (spike S1).

To evaluate the effectiveness of potential candidates to inhibit the binding site, it was assumed that the inhibitor reached the ACE2 receptor first, i.e., before the S viral protein. This molecular docking involves a protein–ligand protocol in which a structural change in the receptor will modify the recognition between ACE2 and RBD of the S1 region. Once the ACE–In docking was made, the next recognition process between the ACE2–In–S1 complex was performed. Therefore, the molecular coupling process was carried out with the module and the conditions described in the validation process of the ligand–protein and protein–protein docking.

### 3.5. Molecular Dynamics Performed for ACE2–In Systems

In this section, the MOE program was used as an interface to prepare the simulation files. However, all simulations were performed with the NAMD 2.13 Multicore processor CUDA [44]. Each of these systems, obtained from the molecular docking between ACE2 and the LQM compounds, were solvated in a periodic box. Periodic conditions allowed for a constant amount of solvent to be maintained in all directions, that is, the number of molecules present in the system was not altered. The dimensions of the box in this case were of the type P1, with distances of 106 Å on each side and angles of 90°. The solvent had sodium chloride (NaCl) with a margin of 6 Å. NaCl was assigned to these simulations as ACE2 is a chloride-dependent protein. The 6 Å margin refers to the minimum distance between the counter ions and the solvent molecules in the system. The complexes were centered, and the axes of the box were aligned. Before carrying out the molecular dynamics, the systems were minimized using an SVL script that was implemented and obtained from repositories of the program to find the global minimum of energy. This script worked by minimizing the system in a progressive way (stepwise), starting with the areas of the highest energy. Minimization was first executed in the solvent, then the protein, and finally in the complete system comprising both the solvent and complexes. All of this was performed with the following specifications: Forcefield Amber10: ETH; eps = 1; Cut-off (10)(12), charges calculations and Gradient: 0.2 RMS. For dynamics, the sample time was 0.5 ps and the time step was 0.002 ps. The total simulation time was 35 ns, of which 5 ns was for heating the system and beginning with the equilibrium process of the system.

### 3.6. Description of the Dynamics of Protein–Protein Systems

In this section, two types of simulations were performed: one that contained ACE2, with and without inhibitors, interacting with the S1 subunit (ACE2–S1), and another that contained ACE2 interacting with the complete trimer of the viral spike. For preparation of the ACE2–S1 complex, the model with PDB code 7KNB was used as a starting point [47]. However, this did not contain the Zn^2+^ cofactor of ACE2. Therefore, the model with PDB code 3SCL was used to model mainly the Zn^2+^ cofactor, and an ACE2 overlay was performed [49].

**Figure 15:**
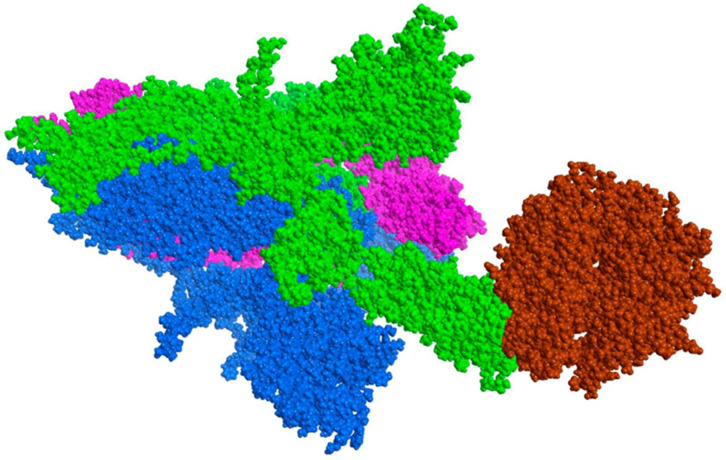
Spike ACE2 system. Sphere model (coarse grain). ACE2 is depicted in brown.

Molecular dynamics studies were performed to analyze the effect of the D614G mutation on the recognition process of the SARS-CoV-2 spike protein with human ACE2. Two systems (with and without mutation) were generated using the native Wuhan spike protein (UniProt: P0DTC2) in complex with ACE2 (UniProt: Q9BYF1). In this case, to have a more precise system, the trimeric spike protein model was used, as at this point it is unknown what impact this mutation may have on a complete system (Figure 15).

The preparation of systems with a greater complexity began with the generation of the missing structures of the ACE2–S1 complex using the Loop Modeler tool of the MOE software. Completed segments were the residuals of the following positions: I 64–83, II 139–153, III 174–189, IV 240–265, V 618–642, VI 674–692, VII 809–817, and VIII 862–855. For this, segments were selected individually, and a search for similarity was carried out, generating a database with the most plausible results. The best five results were selected according to the S score (Score function), and the best value was selected to complete the structure. Conditions for the generation of loops were parameterized on an Amber10: EHT force field and a 12 Å cut-off to homologate the molecular dynamic conditions and try simulate the ideal physiological conditions.

In addition to this, a correction was made for localized errors, such as protonation, which was corrected with the 3D protonate module, completing side chains with the loop modeler module, terminal chain errors with corrections from FF libraries, and adding charges to the atoms with partial charges. The next step was to solvate the complex, adding water as a solvent at periodic conditions. The shape of the periodic box was P1, with dimensions of 134.0 Å on each side and angles of 90° on all of the vertices, thus yielding a periodic cube that was further used to evaluate the dynamic simulations. Neutral charges and partial charges were assigned to atoms with parameters of the mentioned FF. The following step was a minimizing system using the Energy Minimize option with the specifications mentioned above. Finally, using the Dynamics with NAMD option, the following protocol was performed:

- Heating for 1 ns, temperature gradient of 0 K to 300 K.
- Equilibrium of 4 ns at 300 K and 1 atm.
- Production 40 ns at 300 K and 1 atm.

### 3.7. Free Binding Energy Calculations

The Binding Free Energy Estimator (BFEE) is a Python-based software that automates the absolute binding free energy calculations through the alchemical or geometric pathway using molecular dynamics simulations. The degrees of freedom of the protein–ligand (or host–host) system are described by a series of geometric variables (or collective variables), as first described by the Karplus group [40]. In BFEE, generalized geometric variables based on the best fit rotation are used, which, in principle, is available for any protein–ligand complex. Among the parameters that were used for the energetic evaluation were the following:

“Outputenergies, outputtiming, outputpressure, restartfreq, XSTFreq, dcdFreq”: all set to 5000; wrapping all the water molecules; non-water molecules unwrapped; PMETolerance: 10 × 10^−6^; time step: 2.0; rigid bonds: all; rigid tolerance: 0.0001; rigid iterations 400; no flexible cell.

### 3.8. Procedure for the Analysis of “Patches” of the Interaction Surfaces

Protein patches are surface indicators that identify regions of hydrophobicity and the presence of charged regions. These patches are useful, for example, to assess which mutations may lead to better solubility or what the potential sites of interaction may be in protein–protein complexes. The MOE Protein Patch Analyzer panels was used to manipulate, extract, and view the calculated patches. Patches on which the analysis was made were calculated in the sets of each protein. This was done to evaluate if there were any changes that would suggest a decrease in the interaction of both structures, taking the systems without inhibitors as a reference. These contributions were evaluated with each type of interaction, which could be hydrophobic, positive, or negative, as well as the total area and percentage of area of the patch with respect to the total surface. This patch analysis was performed in the following stages: with the reference complex ACE2–S1, and ACE2 with each of the LQM compounds and every system where ACE2–In–S1 is found.

## 4. Conclusions

Upon carrying out the studies corresponding to the interaction between the S1 subunit of the SARS-CoV-2 spike and the human ACE2 receptor, it was found that when the ACE1 inhibitor LQM322 was used, the interaction between both proteins decreased. This, when analyzing the results of the computational analyzes, allowed for determining that the complex interactions and stabilities are affected if the mentioned compound is used. To assert this, a comparison was made with respect to a system without inhibitors in order to know the behavior of the protein complex interaction with respect to time (40 ns). Once compared with the systems where some inhibitors are present, the parameters such as RMSD, RMSF, hydrogen bonds, and interaction patches indicate that the interaction is significantly diminished with the LQM322 compound. It should be mentioned that all of the other LQM compounds also present an alteration in the interaction, but the computational evidence does not allow us to affirm whether it is significant. The LQM304 compound has the least favorable interaction and therefore does not present an alteration during the interaction process between the S1 subunit and ACE2. The less favorable compound interaction with ACE2 corresponds to LQM 304, which has the lowest binding free energy as a consequence of a greater gap (open conformation) in ACE2 during the simulation of molecular dynamics. These compounds displayed a similar behavior when they were tested with the ACE1 isoform. The latter also have centers that allow them to interact through hydrogen bonds, which help stabilize the ACE2–inhibitor complex during the simulation time. In addition to the fact that the complex was stable over time, the interaction energies will allow us to infer that the compounds may have an adequate affinity and that they will be suitable candidates to evaluate using an experimental method. One of the characteristics of the compounds being used is compounds with high reaction yields and low-cost synthesis routes. This agrees with the experimental data reported in the aforementioned study [37]. It is also necessary to mention that from a computational point of view, it is not necessary to use a complete viral spike system, thus allowing for optimizing the calculation time. In this way, it is possible to carry out computational studies that are not as time consuming in these systems in order to propose molecules as candidates that interact with ACE2. With all of the above-mentioned, it is possible to affirm that the LQM319 and LQM322 compounds have a high potential to be tested in in vitro studies in order to evaluate their activity in an experimental model together with the LQM304 compound, which would work as a compound with a low or no inhibitory activity.

## Figures and Tables

**Table 1:** Structure of in-house LQM compounds in two-dimensional representation.

**LQM304**	**LQM318**		**LQM319**
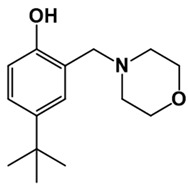	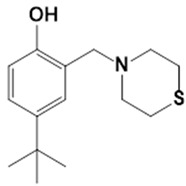		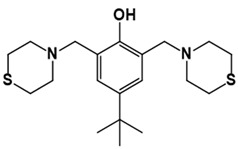
**LQM322**		**LQM324**	
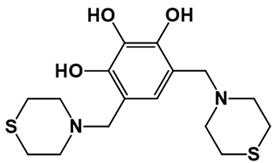		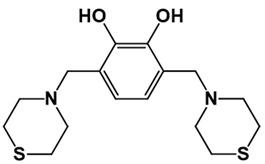	

**Table 2:** Free binding energies for the studied systems computed with BFEE2.

System (ACE2-LQM)	ΔG (kcal/mol)	Deviation ± (kcal/mol)
304	−4.91	0.21
318	−4.98	0.20
319	−5.71	0.41
322	−5.59	0.26
324	−4.89	0.29

**Table 3:** Analysis of hydrophobic, negative, and positive charged patches.

	Area Å2	% ASA
Hydrophobic	419.20	4.87
Negative	224.70	2.62
Positive	204.30	2.38

**Table 4:** Average interaction patches of ACE2 and ACE2 with inhibitors.

	ACE2		304		318		319		322		324	
	AREA	% ASA	AREA	% ASA	AREA	% ASA	AREA	% ASA	AREA	% ASA	AREA	% ASA
Hyd	127.45	0.57	112.25	0.50	66.36	0.30	73.40	0.33	72.85	0.33	63.90	0.29
Negative	150.20	0.67	157.65	0.70	156.35	0.72	148.50	0.66	165.10	0.73	152.15	0.68
Positive	58.60	0.26	0.00	0.00	214.40	0.95	50.50	0.20	163.40	0.74	164.10	0.73

## Supplementary Materials

The following are available online at https://www.mdpi.com/article/10.3390/pathogens10091208/s1, Figure S1: Docking results for each cluster generated from the inhibitor ACE2 receptor.
