# Supplementary material for: Evaluation of Inhibitory Activity In Silico of In-House Thiomorpholine Compounds between the ACE2 Receptor and S1 Subunit of SARS-CoV-2 Spike
Source: Pathogens. 2021 Sep 17;10(9):1208. doi: 10.3390/pathogens10091208 (PMC8468748; doi:10.3390/pathogens10091208)

Supplementary figure 1: Docking results for each cluster generated from the inhibitor ACE2 receptor.

ACE2 receptor's color representations: Red -helixes, yellow - sheets, blue -turns, and white - loops. Color representation of ligands (LQM compounds): Carbon atoms - green; hydrogen atoms – white, oxygen atoms – red, nitrogen atoms - Blue; sulfur atoms - yellow.

The representation of these clusters is made up of 50 docking end positions of each inhibitor studied. The receptor is ACE2, and the inhibitors are the compounds LQM304, LQM318, LQM319, LWM322 and LQM324. Each image is composed as follows: ACE2 and LQM304 (**A**), ACE2 and LQM31 (**B**), ACE2 and LQM319 (**C**), ACE2 and LQM319 (**D**), and ACE and LQM324 (E).

A

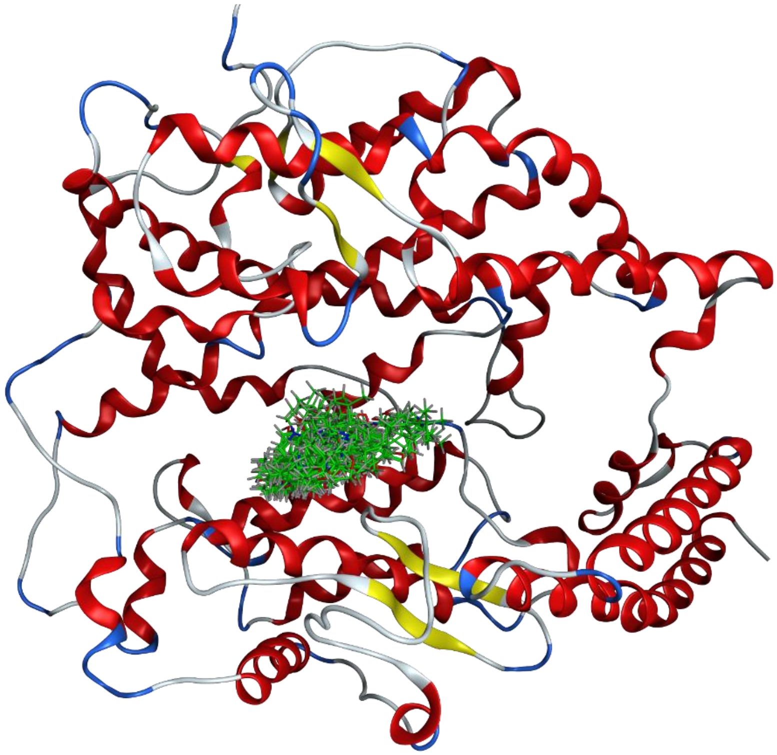

B

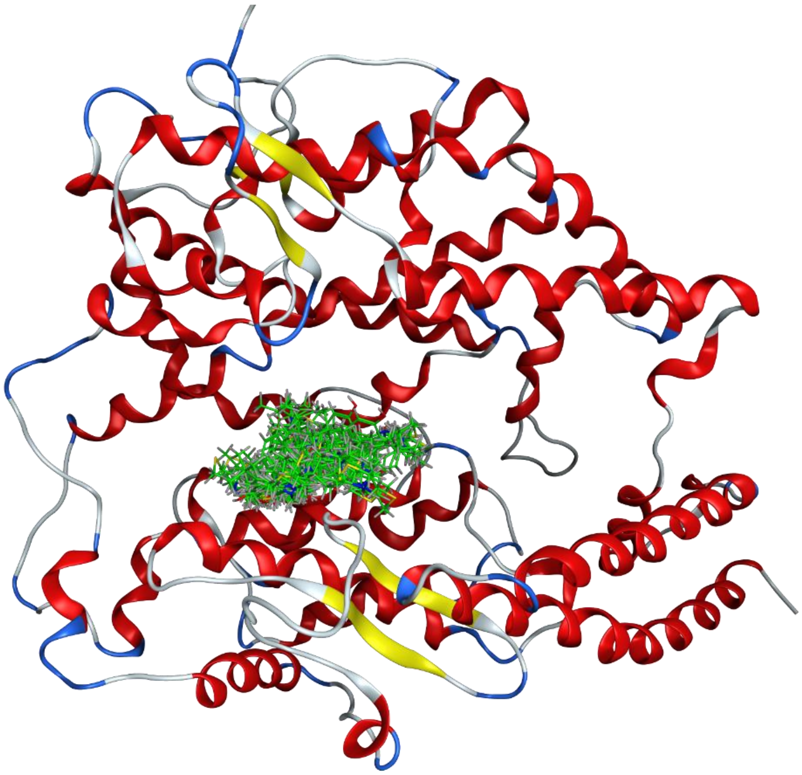

C

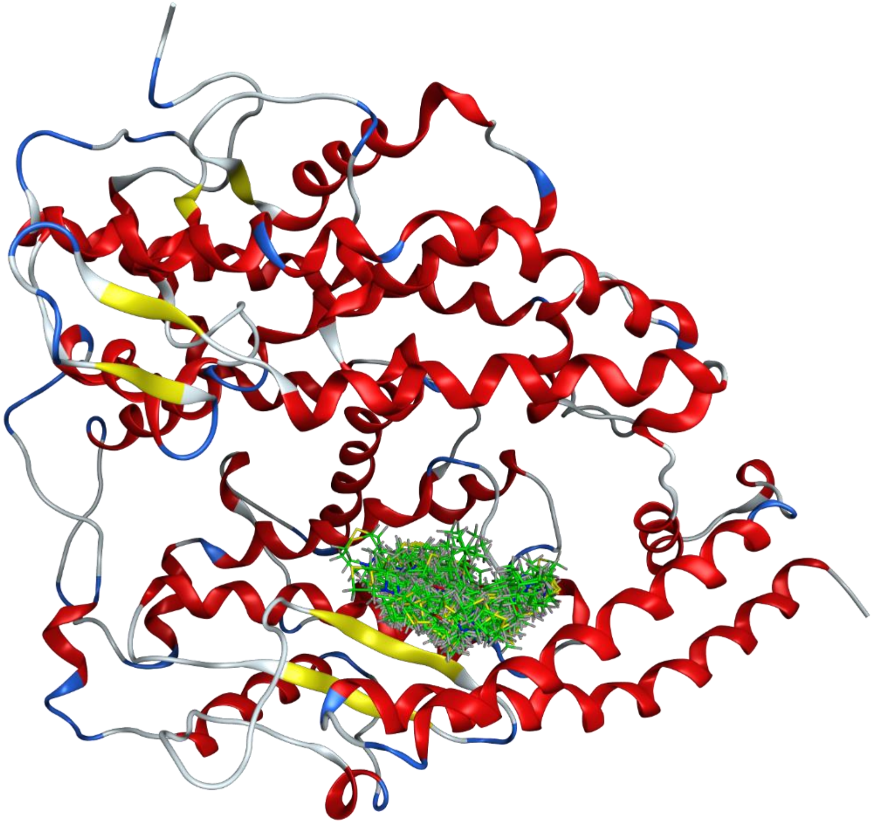

D

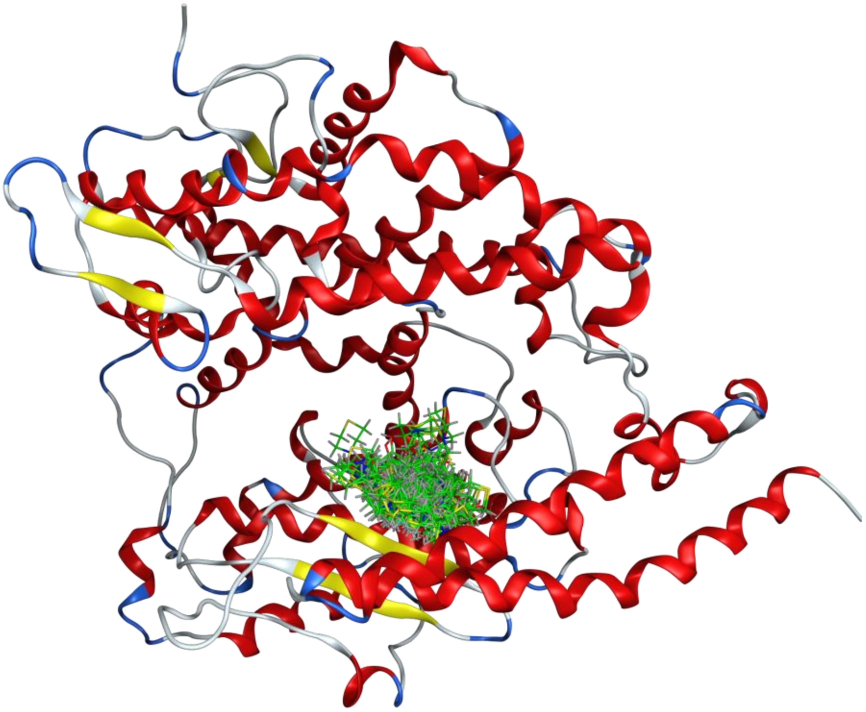

E

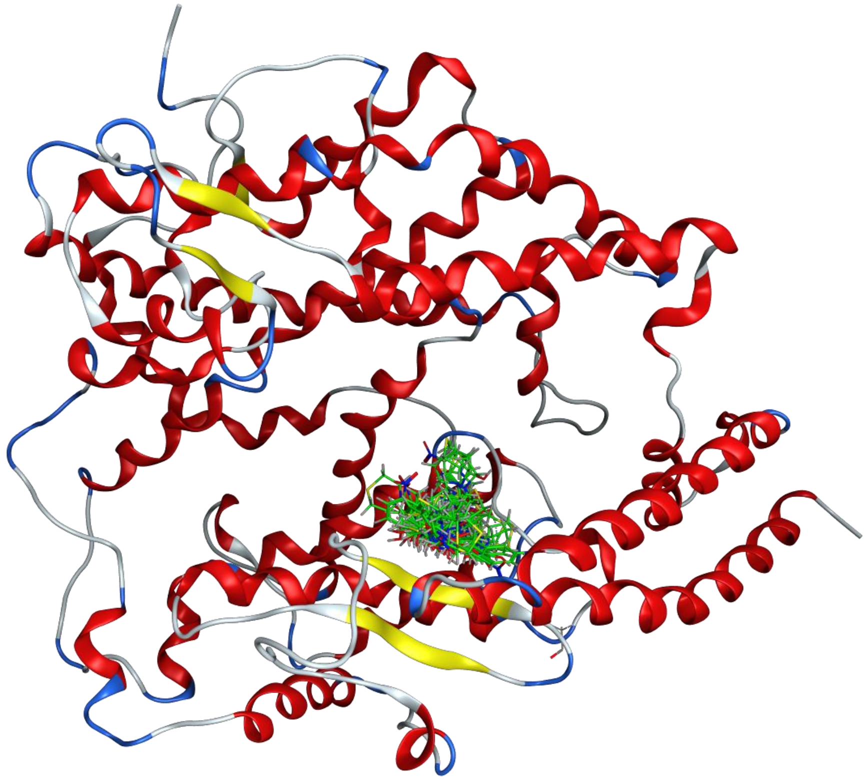

Supplement: Supplementary file 1 [file pathogens-10-01208-s001.zip › pathogens-1351282-supplementary.pdf]
